# Supplementary material for: From graph topology to ODE models for gene regulatory networks
Source: PLoS One. 2020 Jun 30;15(6):e0235070. doi: 10.1371/journal.pone.0235070 (PMC7326199; doi:10.1371/journal.pone.0235070)
Supplement: S1 Appendix — A brief review on both graph models and ODE models is given here. (PDF) [file pone.0235070.s001.pdf]

Supplementary information

Appendix

S1 Basic model of gene interaction

In molecular biology, DNA is transcribed into mRNAs, which in turn are translated into proteins. A gene is a region of the DNA that encodes the corresponding mRNAs and proteins. We say a gene is expressed if it is producing the corresponding proteins, and its expression level refers to the concentration of those proteins.

We consider the transcriptional regulatory networks, which involve the interaction of DNA, mRNA and proteins. In this type of regulatory networks, some proteins are transcription factors (TFs) regulating the transcription of other genes by turning them on or off. For example, gene X produces protein X, which may be a TF that activates

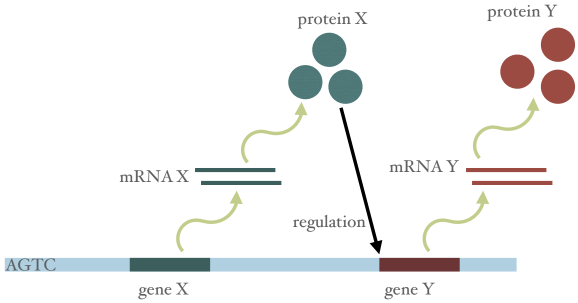

Fig 1S. Direct binding of TFs to the promoter region of the target gene.

gene Y by increasing the transcription rate of gene Y (turning it on). Some TFs are repressors, whose presence decreases the production rate of the genes they regulate (turning them off). See Figures 1S and 2S for an illustration. This model suggests summarizing a gene regulatory network by a directed graph with signed edges, such that the vertices represent genes and the sign on an edge indicates activation or repression.

The gene expression in the transcriptional regulatory networks can be modeled with various levels of granularity. Some use multiple variables for each gene (see, e.g., [1] and [2]), while others use a single variable to represent both the mRNA and the protein concentrations of a single gene (see, e.g., [3]). Some model the interaction between TFs and the promoter region of the target genes directly [3], while others consider intermediate elements called *cis*-regulatory modules (CRMs) between the TFs and the promoters (see [2] and the GeneNetWeaver code [4]).

Furthermore, the transcriptional gene expression can also be affected by protein–protein interaction. One type of such interaction is signal transduction, which most commonly consists of the process of protein phosphorylation catalyzed by protein kinases. The phosphorylated proteins are active TFs, which bind the DNA and regulate transcription (see Chapter 6.3 in [3] and [5]). Another type of such interaction is protein complex forming, where multiple proteins can bind and form a protein complex that act as an active TF.

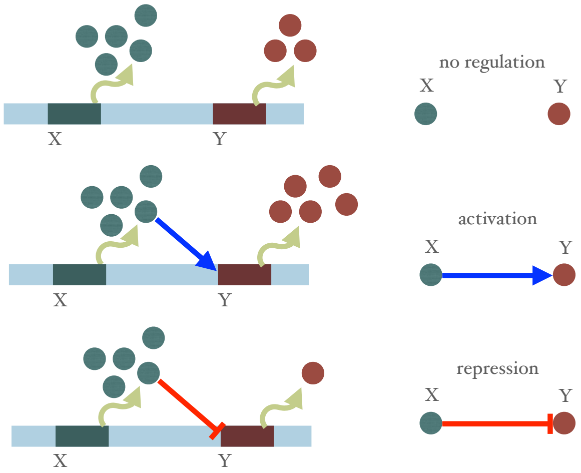

Fig 2S. Types of transcriptional regulation.

References

1. Locke JCW, Millar AJ, Turner MS. Modelling genetic networks with noisy and varied experimental data: The circadian clock in *Arabidopsis thaliana*. J Theor Biol. 2005;234(3):383–393. doi:10.1016/j.jtbi.2004.11.038.

2. Marbach D, Prill RJ, Schaffter T, Mattiussi C, Floreano D, Stolovitzky G. Revealing strengths and weaknesses of methods for gene network inference. Proc Natl Acad Sci USA. 2010;107(14):6286–6291. doi:10.1073/pnas.0913357107.

3. Alon U. An introduction to systems biology: Design principles of biological circuits. CRC press; 2006.

4. Schaffter T, Marbach D. GeneNetWeaver; 2012. Available from: `https://github.com/tschaffter/gnw`.

5. Tyson JJ, Chen KC, Novak B. Sniffers, buzzers, toggles and blinkers: Dynamics of regulatory and signaling pathways in the cell. Curr Opin Cell Biol. 2003;15(2):221–231. doi:10.1016/S0955-0674(03)00017-6.
