# Supplementary material for: From graph topology to ODE models for gene regulatory networks
Source: PLoS One. 2020 Jun 30;15(6):e0235070. doi: 10.1371/journal.pone.0235070 (PMC7326199; doi:10.1371/journal.pone.0235070)
Supplement: S2 Appendix — Specific module generation and parameter ranges in GeneNetWeaver are described here. (PDF) [file pone.0235070.s002.pdf]

Supplementary information

Appendix

S2 Random model for production functions used in GeneNetWeaver

The materials and methods in the main body describes the family of ODE modules for gene regulatory networks, involving *cis*-regulatory modules, used in the GeneNetWeaver code. A main purpose of GeneNetWeaver is to generate random instances of such networks, which includes selecting how to partition the inputs to a given gene into modules and selection of parameter values. The probabilistic model of GeneNetWeaver is summarized in this section.

S2.1 Random module structure

An input to the GeneNetWeaver simulator is a signed, directed graph that specifies for any target gene i, which other genes are inputs that directly influence it, and the signs of the influence. The production function for a target gene i is randomly generated as follows. First, the inputs are partitioned to a random number of modules according to the following process.

1. Inputs are randomly reordered.
2. Module set A is initialized empty.
3. Each input in order is uniformly added to one of the modules in A or a new module, all with probability $\frac{1}{|A|+1}$. The module set A is updated if new modules are created.

The parameter $c_{K:i}$ determines the type of the module $K:i$ (enhancer or silencer). A given input gene in module $K:i$ is an activator if the sign of the gene (in the signed, directed graph) is the same as $c_{K:i}$, and otherwise the given input gene is a deactivator. The software ensures that each module has at least one activator for signed graph input, which means that if for module $K:i$, all the input genes have the same sign, then the $c_{K:i}$ should be the same as the sign of the input genes.

S2.2 Random parameter initialization

This section describes the random initialization of the parameters. Note the truncation is implemented by regenerating i.i.d. random variable until it falls within the desired region, which effectively scales up the probability density restricted to the region to form a proper probability distribution.

- The Michaelis–Menten coefficient is generated by $k_{ij} \sim \mathsf{Unif}([0.01, 1])$.
- The Hill coefficient is generated by $h_{ij} \sim \mathcal{N}(2, 2^2)$ truncated to $[1, 10]$.
- The mRNA degradation rate is $\delta_i^{(\mathrm{m})} = \log(2)/T_{1/2}$, where $T_{1/2} \sim \mathcal{N}(27.5, (7.5)^2)$ truncated to $[5, 50]$. As a result, $\delta_i^{(\mathrm{m})} \in [0.014, 0.14]$.

- The maximum translation rate and the protein degradation rate are both given by the same $\lambda_i = \delta_i^{(\mathrm{p})} = \log(2)/T_{1/2}$, where $T_{1/2} \sim \mathcal{N}(27.5, (7.5)^2)$ truncated to $[5, 50]$ is a realization independent with that for the mRNA degradation rate. Again $\delta_i^{(\mathrm{p})} \in [0.014, 0.14]$.

The basal activation $\alpha_{i,\mathrm{basal}}$ and the vector of absolute module effect parameters $\beta_{K:i}$ are generated using the following procedure. For target gene i:

1. If $N_i = \emptyset$, set $\alpha_{i,\mathrm{basal}} = 1$.
2. Otherwise set $\beta_{K:i} \sim \mathcal{N}(5/8, (1/8)^2)$ truncated to $[1/4, 1]$ for all $K \in \mathcal{S}_i$.
3. If $c_{K:i} = -1$ for all $K \in \mathcal{S}_i$, set $\alpha_{i,\mathrm{basal}} = 1$.
4. If $c_{K:i} = 1$ for all $K \in \mathcal{S}_i$, set $\alpha_{i,\mathrm{basal}} \sim \mathcal{N}(0, (0.05)^2)$ truncated to $[0, 1/4]$.
5. Otherwise $\alpha_{i,\mathrm{basal}} \sim \mathcal{N}(1/2, (1/12)^2)$ truncated to $[1/4, 3/4]$.
6. If $\alpha_{i,\mathrm{basal}} + \sum_{K:\ c_{K:i}=1} \beta_{K:i} < 1$, increase the smallest β therein to reach a maximum activation of 1.
7. If $\alpha_{i,\mathrm{basal}} + \sum_{K:\ c_{K:i}=-1} \beta_{K:i} > 0.25$, increase the smallest β therein to reach a random minimum activation of $\mathcal{N}(0, (0.05)^2)$ truncated to $[0, 1/4]$.
8. Finally, truncate all $\alpha_{i,s}$ to $[0, 1]$.

The resulting $\alpha_{i,s}$'s have a good coverage of the interval $[0, 1]$.
