# Supplementary material for: From graph topology to ODE models for gene regulatory networks
Source: PLoS One. 2020 Jun 30;15(6):e0235070. doi: 10.1371/journal.pone.0235070 (PMC7326199; doi:10.1371/journal.pone.0235070)
Supplement: S3 Appendix — A proof of the equation involving the partial derivatives of the solution of dynamical systems. (PDF) [file pone.0235070.s003.pdf]

Supplementary information

Appendix

S3 Proof of (6)

In this section we focus on the clamped system and write $f = \hat{f}$ for short. If $a = 0$ or $b = 0$, we have $\partial_{t^a h^b} g(0,0) = 0$. If $a \neq 0$ and $b \neq 0$, we have $\partial_{t^a h^b} g(t,h) = \partial_{t^a h^b} \eta_j(t,h)$. So it suffices to show

$$\partial_{t^a h^b} \eta_j(0,0) = \begin{cases} \Delta(i,j,x,\lambda) & \text{if } (a,b) = (q,1), \\ 0 & \text{if } 1 \leq a \leq q-1 \text{ and } b \geq 1. \end{cases}$$

In the following we write $\eta_j \triangleq \eta_j(t,h)$ and $f_l \triangleq f_l(\eta_{p(l)}(t,h), \lambda)$ unless specified otherwise, where $p(l)$ is the set of indices of the variables that the clamped dynamics $\hat{f}_l^{(j)}$ depends on (i.e., the parent set of l). Let $\mathsf{POLY}(s,u)$ denote the set of polynomials of partial derivatives of f_l up to order s for all $l \in \{j\} \cup p(j) \cup \cdots \cup p^u(j)$ (i.e., up to u generations of parents of j).

Lemma 1. *For any t and h,*

$$\partial_{t^a} \eta_j = \begin{cases} f_j & \text{if } a = 1, \\ f'_j f'_{p(j)} \cdots f'_{p^{a-2}(j)} f_{p^{a-1}(j)} + \phi_a & \text{if } a \geq 2, \end{cases} \tag{1}$$

where

$$f'_j f'_{p(j)} \cdots f'_{p^{a-2}(j)} f_{p^{a-1}(j)} = \sum_{\substack{(r_0, r_1, \ldots, r_a) \in [n+m]^{a+1}: \\ r_0 = j, \forall l, r_{l+1} \in p(r_l)}} \left(\prod_{l=0}^{a-2} \partial_{r_{l+1}} f_{r_l} \right) f_{r_{a-1}}$$

and $\phi_a \in \mathsf{POLY}(a-1, a-2)$ is some polynomial of the partial derivatives of f.

Proof. We use induction on a. It is easy to see that $\partial_t \eta_j = f_j$ and $\partial_{t^2} \eta_j = f'_j f_{p(j)}$, so (1) holds for $a = 1, 2$. Now suppose (1) holds for some $a \geq 2$. Then the time derivative of $\psi \triangleq f'_j f'_{p(j)} \cdots f'_{p^{a-2}(j)} f_{p^{a-1}(j)}$ is

$$\partial_t \psi = \psi_1 + f'_j f'_{p(j)} \cdots f'_{p^{a-1}(j)} f_{p^a(j)},$$

for some $\psi_1 \in \mathsf{POLY}(2, a-1)$. Note $\partial_t \phi_a \in \mathsf{POLY}(a, a-1)$. So

$$\partial_{t^{a+1}} \eta_j = f'_j f'_{p(j)} \cdots f'_{p^{a-1}(j)} f_{p^a(j)} + \psi_1 + \partial_t \phi_a$$

and $\psi_1 + \partial_t \phi_a \in \mathsf{POLY}(a, a-1)$. Induction completes the proof. □

It is then easy to see that for $a \in [q-1]$,
$\partial_{t^a} \eta_j \in \mathsf{POLY}(a-1, a-1) \subseteq \mathsf{POLY}(q-2, q-2)$, which does not depend on h when $t = 0$. Hence $\partial_{t^a h^b} \eta_j(0,0) = 0$ for any $a \in [q-1]$ and $b \geq 1$. For $a = q$, when $t = 0$, the only terms in $\partial_{t^a} \eta_j$ that depend on h are those in $f'_j f'_{p(j)} \cdots f'_{p^{a-2}(j)} f_{p^{a-1}(j)}$ with paths connecting i to j. So $\partial_{t^q h} \eta_j(0,0) = \sum_{(r_0, r_1, \ldots, r_q) \in \mathcal{P}_{ij}^q} \prod_{l=0}^{q-1} \partial_{r_{l+1}} f_{r_l}(x, \lambda)$.
