# Supplementary material for: From graph topology to ODE models for gene regulatory networks
Source: PLoS One. 2020 Jun 30;15(6):e0235070. doi: 10.1371/journal.pone.0235070 (PMC7326199; doi:10.1371/journal.pone.0235070)
Supplement: Attachment — Submitted filename: Review PloS One 2020.pdf [file pone.0235070.s004.pdf]

Review of PONE-D-20-02246

March 6, 2020

The paper under review is unfocused and rather weak. The main purpose seems to be to define carefully an ODE model called GeneNet Weaver that has been used to to produce data used in DREAM challenges. Then the main result seems to be observation in three lines that the ODE's generated by this model have monotone dependence on their arguments and therefore are consistent with a network graphical model with signed edges.

However, there is insufficient justification why this class of models reflects biological reality. For instance, the module 1 uses product between all activators and deactivators which assumes that all activators must be bound for transcription to happen. This is not true in many promoter regions where either activator can activate the gene. If these modules can be then onsidered independently, this must be explained and stated.

In line 205 a statement about a sum being considered 1 vs zero is puzzling, and left hanging without any explanation. Yet this seems to be. a crucial point of the discussion about relationship between modules 2 and 3.

There is no comparison with older similar models of gene regulation based on statistical mechanics and occupancy or promoters. In particular, line of papers

- Ackers GK, Johnson AD, Shea MA., Quantitative model for gene regulation by lambda phage repressor., Proc Natl Acad Sci U S A. 1982 Feb;79(4):1129-33
- Shea MA, Ackers GK., The OR control system of bacteriophage lambda. A physical-chemical model for gene regulation., Mol Biol. 1985 Jan 20;181(2):211-30.

should be cited and discussed. In particular,

- Gedeon, Mischaikow K, Patterson K, Traldi E., When activators repress and repressors activate: a qualitative analysis of the Shea-Ackers model. Bull Math Biol. 2008 Aug;70(6):1660-83. doi: 10.1007/s11538-008-9313-6. Epub 2008 Jul 22.

has shown that the Shea-Ackers type model does not have to have the monotonicity property that is claimed in this paper for GeneNet Weaver models.

Discussion on what assumptions on GeneNet Weaver models guarantee the monotonicity must be made.

It is not clear what the case study communicates. Artificial data is generated and then fit with ODE models. This has no bearing on biology.

I recommend either a rejection or a major revision for this paper.
