# Supplementary material for: From graph topology to ODE models for gene regulatory networks
Source: PLoS One. 2020 Jun 30;15(6):e0235070. doi: 10.1371/journal.pone.0235070 (PMC7326199; doi:10.1371/journal.pone.0235070)
Supplement: Attachment — Submitted filename: response.pdf [file pone.0235070.s005.pdf]

May 24, 2020

Dear Dr. Hernandez-Lemus:

I am pleased to submit a revision of our original research article entitled "From graph topology to ODE models for gene regulatory networks" by Xiaohan Kang, Bruce Hajek, and Yoshie Hanzawa for consideration for publication in PLOS ONE. Please find our responses to the reviewer's and the editor's comments below.

Reviewer #1

Comments:

1. *Overall, most given explanations are too brief and for this reason the ideas introduced are difficult to understand. The paper should be extended - at the moment it is anyway quite short.*
   Importantly, there is no deeper discussion of the obtained results (also a discussion section is missing). At least 3-4 of discussion should be added.
   The paper is currently in a draft stage and required more work.
   Introduction: The related literature is not properly cited or discussed. Please add more background information about this. In total, about 40 papers should be cited (currently only 18 are cited).
   Response: We thank the reviewer for this comment and have significantly improved the discussion on the background and the related literature. The number of pages is now 34 compared to 22 in the original submission. The number of references is now at 41 compared to 18 in the original submission. We have also added three discussions in the Discussion section.

2. *It is unclear to me how the authors define a 'gene regulatory network'. Frequently this is done wrong, as discussed here*
   PMID: 25364745
   Importantly, a gene networks refer to all possible types of molecular interactions, including transcriptional regulation or protein interaction. If it would only refer to the former it would be a transcriptional regulatory network.

Furthermore, the modeling perspective of such networks has been discussed in detail in PMID: 25221572

Response: We thank the reviewer for the comment and the references. We have clarified the definition of a gene regulatory network at the beginning of the introduction (page 2 of the revised manuscript with track changes). Our general ODE models in the Materials and Methods section include all types of molecular interactions, as evident in the example in Fig 2.

3. *It is unclear of GeneNetWeaver is essential for the analysis of if different tools could be used, e.g.,*

   PMID: 16438721

   This point should be made clear.

   GeneNetWeaver is an old model. Is there no newer development in this area?

   Response: We thank the reviewer for the comment on clarification of GeneNetWeaver and for the reference. GeneNetWeaver is a popular tool for generating biologically plausible gene expression data, on which the GeneNetWeaver ODE model in Materials and Methods section is based on. The model is highly versatile because it can incorporate protein–protein interactions, signal transduction, protein complex, etc. Although GeneNetWeaver is old, it is still up-to-date and has been used in simulating bulk expression data and more recently extended to single-cell expression data (see page 3 of the revised manuscript with track changes). The GeneNetWeaver ODE model is used as an example to demonstrate the analysis of constant sign property and is shown to satisfy global constant sign property. Same analysis applies to general ODE models in Materials and Method section as well, and can be even extended to other gene regulatory network models like the fixed-point model in Van den Bulcke et al. (PMID: 16438721) and Bayesian network models (see discussion on pages 31–33 of the revised manuscript with track changes).

4. *Fitting results: This section is entirely unclear. I guess a regression is performed? What is the used sample size? What is R^2? What regularization has been used? How is the fit evaluated? How is dealt with overfitting?*

   Response: We thank the reviewer for pointing out the missing details in the curve fitting results. We have added technical details of the fitting, a table of the coefficients of determination, and some discussion on regularization. See pages 29 and 30 of the revised manuscript with track changes.

5. *Conclusions: The insights provided are shallow.This problem related back to my points I made above.*

   Response: We thank the reviewer for the feedback and hope the reviewer would appreciate the new discussions, background information, related literature, clarifications, and new notion of infinitesimal constant sign property and its relation to the original sum–product constant sign property in the revision.

6. *What does the CSP mean biologically?*

   Response: We thank the reviewer for this important question. CSP identifies a set of conditions under which genes are regulated in a consistent way. GCSP, which identifies the set to be all conditions, indicates a level of homogeneity of the gene regulatory network for different cell types and different external conditions. See the new discussion on page 33 of the revised manuscript with track changes.

7. *The paper mentions no word that usually GRN do have many more than 5 genes? Can one extend the model to 100 genes?*

   Response: We thank the reviewer for pointing this out. Both the GeneNetWeaver ODE model and the general ODE model in the Materials and Methods section apply to gene regulatory networks with an arbitrary number of genes, and the analysis of CSP applies to such models too. Note that a network of 100 genes is less likely to satisfy GCSP than a network of 5 genes because more regulatory interactions need to be checked.

Reviewer #2

Summary: *The paper under review is unfocused and rather weak. The main purpose seems to be to define carefully an ODE model called GeneNetWeaver that has been used to to produce data used in DREAM challenges. Then the main result seems to be observation in three lines that the ODE's generated by this model have monotone dependence on their arguments and therefore are consistent with a network graphical model with signed edges.*

Response: We thank the reviewer for the careful reading. We clarify in the revision that the main message of the paper is the concept of constant sign property that connects ODE models and graph models. GeneNetWeaver is used as an example where the constant sign property is satisfied globally regardless of the internal or external conditions. The constant sign property also applies to the general ODE model in Materials and Methods and its

generalization to other gene regulatory networks is discussed in the Discussion section. We believe the constant sign property is crucial in understanding the relation between ODE models and graph models for gene regulatory networks.

Comments:

1. *However, there is insufficient justification why this class of models reflects biological reality. For instance, the module 1 uses product between all activators and deactivators which assumes that all activators must be bound for transcription to happen. This is not true in many promoter regions where either activator can activate the gene. If these modules can be then considered independently, this must be explained and stated.*
   Response: We apologize for the confusion. In GeneNetWeaver ODE models the activation of a single module requires all its activators to be bound. However transcription factors can regulate a target gene through multiple modules such that each transcription factor activates the target gene to a certain degree. This does not emulate an ideal OR gate but is still versatile enough for wide usage (see pages 3 and 4 of the revised manuscript with track changes).

2. *In line 205 a statement about a sum being considered 1 vs zero is puzzling, and left hanging without any explanation. Yet this seems to be a crucial point of the discussion about relationship between modules 2 and 3.*
   Response: We apologize for the confusion. The relationship between Type 2 and Type 3 modules is rephrased on page 17 of the revised manuscript with track changes.

3. *There is no comparison with older similar models of gene regulation based on statistical mechanics and occupancy or promoters. In particular, line of papers*
   Ackers GK, Johnson AD, Shea MA., Quantitative model for gene regulation by lambda phage repressor., Proc Natl Acad Sci U S A. 1982 Feb;79(4):1129-33
   Shea MA, Ackers GK., The OR control system of bacteriophage lambda. A physical-chemical model for gene regulation., Mol Biol. 1985 Jan 20;181(2):211-30.
   should be cited and discussed. In particular,
   Gedeon, Mischaikow K, Patterson K, Traldi E., When activators repress and repressors activate: a qualitative analysis of the Shea-Ackers model.
   Bull Math Biol. 2008 Aug;70(6):1660-83. doi: 10.1007/s11538-008-9313-6. Epub 2008 Jul 22.
   has shown that the Shea-Ackers type model does not have to have the monotonicity

property that is claimed in this paper for GeneNet Weaver models.

Discussion on what assumptions on GeneNet Weaver models guarantee the monotonicity must be made.

Response: We thank the reviewer for the two new references (the first one was already cited). Both Shea–Ackers model and the GeneNetWeaver model fall into the general ODE model in the Materials and Methods section, and they both generalize Hill kinetics to multi-input scenarios. Shea–Ackers model considers arbitrary control state energy and may not satisfy the global constant sign property as noticed in Gedeon et al. The GeneNetWeaver model characterizes the regulatory effect by the sum of multiple special Shea–Ackers type modules. Due to the additivity of the modules and the additivity of the control state energy, the GeneNetWeaver model can be shown to satisfy global constant sign property. We have included the comparison on pages 5, 16, 17, and 23 of the revised manuscript with track changes.

4. *It is not clear what the case study communicates. Artificial data is generated and then fit with ODE models. This has no bearing on biology.*

   Response: We apologize for the confusion on the case study. Assuming the widely used GeneNetWeaver model is biologically plausible, this case study investigates whether the ODE model and the graph model can be recovered from the expression data or not. Such study on synthetic data is useful because biological data often lack a ground truth that is necessary for performance evaluation. We hope the reviewer would appreciate some clarification on the case study on pages 29 and 30 of the revised manuscript with track changes.

Editor's comment:

The reviewers point out to the need to deepen the level of description and clarifying the concepts behind your analysis and results. Compare the performance of the present method with different methods, as well as highlighting the novelty and usefulness of this approach.

Response: We thank the editor for the comment. We have revised the manuscript according to the reviewers' comments with numerous clarifications and discussions, showing the novelty and importance of the constant sign property proposed in this manuscript. We also added a different notion of constant sign property and include a proposition on the relation between the two notions.

Sincerely,

Xiaohan Kang, PhD

Postdoc, Coordinated Science

Laboratory and Department of

Electrical and Computer Engineering

University of Illinois at

Urbana–Champaign
